# Differential Tick Salivary Protein Profiles and Human Immune Responses to Lone Star Ticks (*Amblyomma americanum*) From the Wild vs. a Laboratory Colony

**DOI:** 10.3389/fimmu.2019.01996

**Published:** 2019-08-28

**Authors:** L. Paulina Maldonado-Ruiz, Lidia Montenegro-Cadena, Brittany Blattner, Sapna Menghwar, Ludek Zurek, Berlin Londono-Renteria

**Affiliations:** ^1^Medical/Veterinary Entomology Laboratory, Department of Entomology, Kansas State University, Manhattan, KS, United States; ^2^Vector Biology Laboratory, Department of Entomology, Kansas State University, Manhattan, KS, United States; ^3^Department of Pathology and Parasitology, CEITEC Center for Zoonoses, University of Veterinary and Pharmaceutical Sciences, Brno, Czechia

**Keywords:** *Amblyomma americanum*, lone star tick, salivary proteins, human immune response, antibodies, seasonal response

## Abstract

Ticks are a growing concern to human and animal health worldwide and they are leading vectors of arthropod-borne pathogens in the United States. Ticks are pool blood feeders that can attach to the host skin for days to weeks using their saliva to counteract the host defenses. Tick saliva, as in other hematophagous arthropods, contains pharmacological and immunological active compounds, which modulate local and systemic immune responses and induce antibody production. In the present study, we explore differences in the salivary gland extract (SGE) protein content of *Amblyomma americanum* ticks raised in a laboratory colony (CT) vs. those collected in the field (FT). First, we measured the IgG antibody levels against SGE in healthy volunteers residing in Kansas. ELISA test showed higher IgG antibody levels when using the SGE from CT as antigen. Interestingly, antibody levels against both, CT-SGE and FT-SGE, were high in the warm months (May–June) and decreased in the cold months (September–November). Immunoblot testing revealed a set of different immunogenic bands for each group of ticks and mass spectrometry data revealed differences in at 19 proteins specifically identified in the CT-SGE group and 20 from the FT-SGE group. Our results suggest that differences in the salivary proteins between CT-SGE and FT-SGE may explain the differential immune responses observed in this study.

## Introduction

Ticks are obligate blood-feeding ectoparasites of a wide range of vertebrates and are important vectors of human and animal pathogens (1). Hard ticks (Ixodidae) feed once in each developmental stage for a prolonged period by deeply penetrating the epidermis, forming a pool of blood, and causing great damage to the host skin that could last from several days to weeks (2, 3). The saliva composition of the tick and other hematophagous arthropods is complex and includes vasodilators, anti-coagulation compounds, and platelet aggregation inhibitors (2, 4). In ticks, saliva composition changes at different time points (5, 6). Arthropods use saliva to counteract the response against injury caused during the process of blood feeding (piercing and tearing) that trigger host defense mechanisms as well as to facilitate the process of obtaining the blood. Evidence suggests that pathogens may take advantage of the immunomodulatory properties of the arthropod saliva to establish infection (7–9). Arthropod saliva modulates the transmission of pathogens either directly by enhancing pathogen invasion (10, 11) or indirectly by modulating host immune responses (12, 13).

Also, salivary proteins may induce potent antibody responses that can be associated with the intensity of exposure to arthropod saliva and can be used as a proxy to measure the degree of vector-host interaction (14, 15). The presence of anti-tick saliva antibodies in human serum can be measured by enzyme-linked immunosorbent assay (ELISA) as a biologic marker of tick exposure with epidemiologic applications. For instance, in the state of California, Lane et al. found a significant correlation between the antibodies to *Ixodes pacificus* and *Borrelia burgdorferi* (16). Previous studies also suggest that the vertebrate immune system exert immunological pressure on the arthropod (2). Specifically, studies report that arthropods may display differences in the composition of their saliva when exposed to different hosts (17, 18). The development of immunity against specific salivary proteins may impair feeding (11, 19, 20), thus it is expected that arthropods try to induce lower antibody levels against proteins that are special for blood feeding. Although the development of strong immunity against salivary proteins is rarely seen in nature (21), this characteristic is being exploited to develop anti-tick vaccines (22).

The lone star tick, *Amblyomma americanum*, is widely distributed across the Southeast and Midwest of the USA and have begun to spread to the central plains of the USA and to Canada (23, 24). This species is an important vector of *Francisella tularensis, Ehrlichia sp*., and other pathogens (25, 26) and has been associated with triggering red meat allergy (27). Previous studies suggest a great diversity among *A. americanum* specimens collected in different states across the US. Also, among ticks raised in colony vs. the ones found in the wild (28). In this study, we tested the hypothesis that field collected *A. americanum* ticks have greater diversity in their salivary protein content than those raised in a colony for several generations, thus inducing different immune responses in the vertebrate hosts when feeding. Our preliminary approach was to explore the differences between ticks raised in a laboratory colony compared to those collected from the field by characterizing (a) the salivary gland extract protein content, (b) antibody levels against the salivary glands extract, and (c) the *in vitro* effect of tick salivary gland content on human cells using specific markers for inflammation and/or cell damage. Our aim was to identify important proteins subject to immunological pressure in the field and to detect specific salivary proteins that could be used to evaluate arthropod host interaction. Our preliminary results revealed important differences in the salivary content of ticks from the field that could potentially have an impact in pathogen transmission.

## Materials and Methods

### Tick Specimens

Laboratory-reared colony non-fed *A. americanum* female adult ticks (CT) were obtained in 2017 from the Department of Entomology and Plant Pathology tick rearing facility at Oklahoma State University (Stillwater, OK). This tick colony was started in 1976 with engorged females collected in Oklahoma. Engorged females are introduced every 2 years in approximately equal numbers to mated colony females. All adult females are reared on sheep and kept at 94–96% humidity, and on a 12:12-hr light:dark cycle. All CT requested for this study were more than 2 months old (based on molting time). Field non-fed-questing *A. americanum* female adult ticks (FT) (unknown molting date) were collected from northeastern Kansas (Konza Prairie Biological Research Station) during summer in 2017 and 2018 using the cloth flagging method. Flags were made by attaching a 95 cm by 70 cm flannel cloth to a wooden utility handle (120 cm). Flagging was carried out by dragging the cloth over the grass area on the edge of the forest for 3–4 m. Ticks were removed from the cloth and placed in glass containers stored in a cooler with high humidity (>90% RH) until arrival to the laboratory. *A. americanum* females were identified by distinct morphological characteristics of ticks found in the state of Kansas (29). Main morphological features included; long palps and ornate scutum typical of genus *Amblyomma* and the distinct white spot located on the edge of scutum; characteristic of the *A. americanum* female (30). All ticks were stored at 4°C in 100% R.H (relative humidity) until used for the experiments.

### Salivary Gland Extraction and Antigen Preparation

Ticks were surface sterilized using 0.5% sodium hypochlorite, 70% ethanol, and washed with sterile water before dissections. Ticks were immobilized on sterile dental wax and sterile phosphate buffer saline (PBS) was added. Tick dorsal integument was removed by a surgical scalpel and salivary glands were extracted. Dissections were performed initiating from anterior part of ticks to minimize the risk of contamination by avoiding midgut in posterior end. Salivary glands were removed and rinsed with PBS prior pooling based on the origin (FT and CT). Salivary gland extract (SGE) was obtained through freeze-thaw cycles (−80°C and 27°C/3 cycles). Protein concentration was measured using a NanoDropTM 2000 spectrophotometer (Thermo Fisher Scientific). Twenty ticks (10 FT and 10 CT) were selected for salivary gland dissections.

### Human Blood Sampling

The protocols followed in the study were reviewed and approved by IRB #1206 from Kansas State University. Blood samples were obtained from 36 adult volunteers residing in Manhattan, Kansas thought finger prick. Blood samples were collected in filter papers (Whatman 903). Samples were collected in the summer 2018 (May–June), and only 27 of those could be followed in the fall (September–November). Blood was collected by the finger prick method as reported elsewhere (31) and blood drops were placed in. Dried blood spots in filter paper were eluted in PBS for further testing. At enrollment, tested individuals were provided with a questionnaire to gather information about age, gender, use of repellant, travel, and outdoor activities.

### ELISA Antibody Levels Testing

Antibody levels were determined by an indirect ELISA following our previous published methodology (15). Total SGE from CT and FT were used as an antigen in the ELISA-based test conducted in 96-well ELISA plates (Nunc-Maxisorp, Nalgene Nunc International, Rochester, NY, USA). Plates were coated with 50 μl/well of tick SGE at a final concentration of 1 μg/ml prepared in 1X PBS and incubated overnight at 4°C. Blood from filter paper was eluted as follows, the fourth part of every dry blood spot (DBS) circle was eluted in 500 μl of PBS 1X overnight at 4°C. Plates were rinsed twice with washing buffer (1X PBS-0,05 % tween 20) and treated with blocking buffer (non-fat dry milk 2%, Tween 20 0.05%, 1X PBS) for 1.5 h at 37°C. Plates were then rinsed twice with washing buffer, and 50 μl/well of the eluted blood (1:50 diluted in blocking buffer) was incubated for 1.5 h at 37°C. After three washes, plates were incubated with horseradish peroxidase-conjugated goat anti-human IgG (Abcam, Ab81202) in a 1:1,000 dilution in blocking buffer. The colorimetric development was obtained using tetra-methyl-benzidine (TMB) (Gene-Script, Piscataway, NJ, USA). The reaction was stopped with 2N sulfuric acid and absorbance was measured at 450 nm. Two controls were included on each plate: (1) negative control: two wells with antigen and without sample as control for non-specific induction of color, caused by any of the reagents used in the test (2) positive control: 1 control per plate (same sample) to test plate variation and normalize OD (optical density) values as described elsewhere (15, 32). IgG antibody levels are reported as ΔOD = Average patient OD value (duplicate) minus the negative/blank control OD.

### Tick SGE Protein Electrophoresis and Immunoblotting

For protein separation, the same amount (10 μg) of FT-SGE and CT-SGE were seeded in two miniprotean TGX gels (Bio-Rad) by duplicate. Precision Plus Protein™ 10–250 kDa Kaleidoscope™ (Thermo Fisher) was used as molecular weight marker and the gel was exposed to 150 V for 45 min. One gel was then washed with PBS and the proteins were visualized using the Pierce Silver Stain kit (Thermo Scientific) according to the manufacturer's instructions. A second gel was used for transferring proteins into a PVDF blotting membrane, trans-blot turbo (Bio-Rad). PVDF membrane with the tick salivary proteins was incubated overnight at 4°C with a 1:100 dilution of a pool of eluted blood samples (*n* = 10) from filter paper (as described above) using ELISA blocking buffer. After three washes with ELISA washing buffer, the membrane was incubated 2 h at room temperature with horseradish peroxidase-conjugated goat anti-human IgG (Abcam, Ab81202) in a 1:1,000 dilution in blocking buffer. The membrane was then washed three times with 1X PBS and incubated with TMB for membranes (Thermo Fisher) and the reaction was stopped with deionized water until the desired color was reached. Reactive proteins were measured using; My Image analysis software using the immunoblot picture as described elsewhere (15). Bands were identified and cut from the stained gel (sizes of ~10 and 20 kDa) and sent in duplicates for sequencing by UPLC-MS/MS.

Gel bands corresponding to immunogenic bands observed in the immunoblotting were sent for sequencing. Briefly, two independent samples for every band were obtained and analyzed. Every protein name was searched in the UNIPROT database and a blast search was performed. Gene ontology was also analyzed using UNIPROT. Features like protein weigh, identity with *A. americanum* and cell localization were recorded for all proteins. From the top 100 identified proteins using mass spectrometry (MS), 39 proteins were selected in total according to the occurrence probability >1.066 and expected weight.

### Cell Lines

The Monocyte-like U937 (ATCC), endothelial HUVEC and neuroblastoma SH-SY5Y (Sigma-Aldrich) cell lines were used in this study to assess inflammation and cell damage. To evaluate the effect of SGE on immune on phagocytic cells (33) we used the mononuclear derived U937 cells were cultured in RPMI 1640 medium supplemented with 10% Fetal Bovine Serum (FBS) and penicillin/streptomycin 1%. Also, to evaluate the effect of endothelial tissue (34), we used HUVEC cells (Millipore-Sigma) cultured in endothelial cell growth medium following sellers' instructions. Since a significant number of viruses transmitted by ticks are neurotropic and arthropod salivary protein may disrupt the nerve-blood barrier, we used the SH-SY5Y neuroblastoma cells to evaluate the effect of SGE on neuronal physiology as described previously (35, 36). SH-SY5Y cells were cultured in DMEM medium supplemented with 15% heat-inactivated FBS and 1% Penicillin/streptomycin.

### Cytokine Induction by Tick SGE

Cells were seeded in 24-well plates, 24 previous to the experiment. Cells were exposed to 1 μg/ml of SGE from either FT or CT an incubated for 24 h at 37°C and 5% of CO_2_. After incubation, the cell pellet was collected and lysed using the RNA lysis buffer (Zymo Kit, cat R1055). Total RNA was extracted using Quick-RNA Kits (Zymo Research) and following manufacturer's instructions. RNA was used to produce cDNA using the RT2 First strand synthesis kit (Qiagen) and kept at −20°C until used.

In HUVEC cells, we measured the gene expression of fibronectin 1 (*FN1*) and thrombospondin (*TSP1*) (37, 38). For the SH-SY5Y cell line, we tested the *CASPASE 3, enolase 2*, Toll-interacting protein (*TOLLIP*), and myeloid differentiation factor-88 adaptor protein (*MyD88*) genes previously associated with injury/damage and immunity in these cells (39–42) (Supplementary Table S1). In addition, cytokine gene expression in the U937 cell line (macrophages) was evaluated using the Applied Biosystems® TaqMan® Array Human Cytokine Network 96-well Plate (Thermo Fisher) following the manufacturer's instructions. Afterward, genes that were found up-regulated were tested further to confirm the results. For this, we used a set of previously published primers: Interleukine (IL) IL8, IL-18, IL12a, IL1B and Tumor Necrosis Factor alfa (TNFα) (43–45), C-C Motif Chemokine Ligand 5 (CCL5), IL-10 (46), and Interferon gamma (IFNγ) (47). These reactions were performed using the PowerUp™ SYBR™ Green Master Mix (Thermo-fisher) in the Quantstudio 3 (Applied Biosystem) PCR thermocycler following manufacturer's instructions.

### Data Analysis

The difference between two independent groups (i.e., antibody levels between males vs. females, fall vs. summer) was determined using the Mann-Whitney test with a *p* < 0.05. Correlation between to independent parameters was done using Spearman correlation method. Paired analysis (i.e., IgG antibodies in the summer vs. fall) we used Wilcoxon-matched pair test. Fold gene expression was calculated by the relative quantification 2^−ΔΔ*ct*^ method using the β2 macroglobulin as the housekeeping gene and cells without treatment as control. To test for statically significant differences (*p* < *0.05*) between the independent groups Statistical analysis was performed using GraphPad Prism, version 8.1 (GraphPad Software Inc., La Jolla, CA).

## Results

### Antibody Responses Against Whole SGE Proteins

To evaluate human immune responses against tick saliva, we collected blood samples from 36 volunteers living in Manhattan, KS, who reported no current illness at the time of sampling. The study sample was composed of 20 women and 16 men, with an age average of 39 years old (from 22 to 69 years) (Table 1). We collected samples from all 36 individuals in summer 2018, however, only 27 participants volunteered for a second sample in the Fall. Only 3 individuals tested (2 females, 1 male) reported to have traveled outside the US during summer, however, all volunteers reported to participate in outdoor activities during this time of the year and only 11 participants reported not using repellent during these activities.

**Table 1:** Summary of healthy volunteers participating in our study (individuals with outdoor activity associated with tick habitat).

	Males^*^	Females^*^	**Total**
Summer	16 (7)	20 (9)	36
Fall	12 (5)	15 (6)	27

*Outdoor activities included hiking, gardening, camping and yard work*.

* *Individuals who traveled outside the US during summer (2 females, 1 male). Only 1 male and 1 female were tested again for fall*.

Comparison of the IgG antibody levels against each SGE type (FT vs. CT) showed significantly higher antibody levels against the CT-SGE than against the FT-SGE (Mann-Whitney test, *p* = 0.0094). In addition, antibody levels against both SGE antigens were significantly higher in the summer months than in the Fall (Mann-Whitney test, *p* < 0.05), but no significant differences were observed when comparing antibody levels between males and females, or after comparing people using repellent or not (Mann-Whitney test, *p* > 0.05) (Figures 2A–D).

### Salivary Profiles and Immunogenic Proteins in SGE From CT and FT

**Figure 1:**
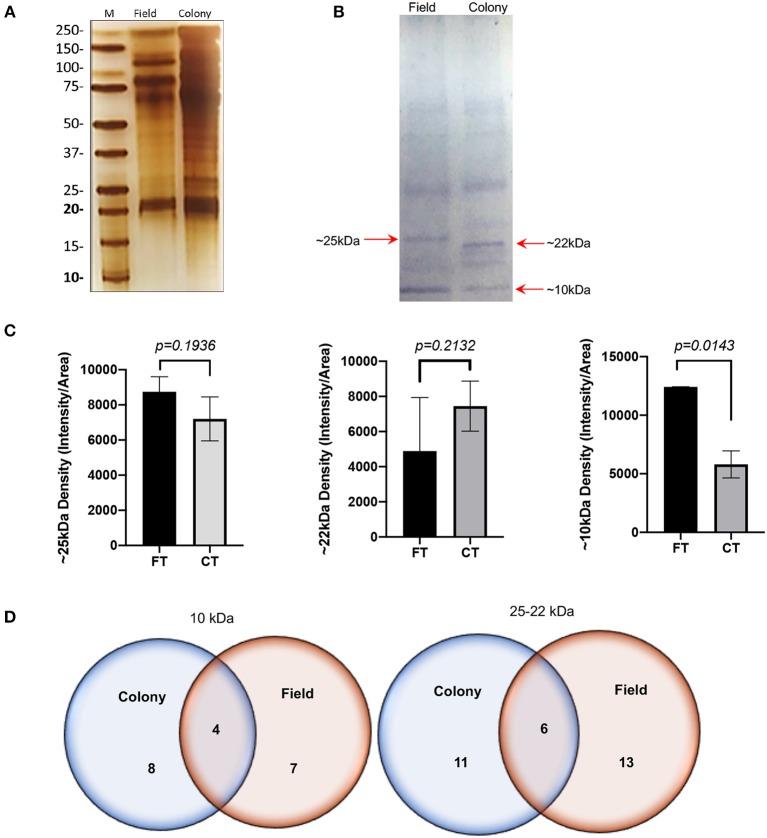
SGE protein analysis Colony and Field. **(A)** Protein SDS-PAGE (silver stain). **(B)** Immunoblot using human samples from healthy volunteers. **(C)** Intensity comparison of immunogenic bands from the immunoblot, and **(D)** Schematic representation of proteins identified by mass spectrometry. Figure displaying the median with interquartile range. Significance evaluated by the Mann-Whitney test with a p < 0.05.

The SDS-PAGE analysis showed discrete differences in the SGE protein content from the CT-SGE and FT-SGE (Figure 1A). In general, higher protein content was observed in the CT-SGE in spite that equal amount of protein was loaded on each lane. However, the immunoblot testing the reactivity of human samples against both SGE revealed a significant strong reactivity with a band around ~25 kDa in the FT- SGE and a band around ~ 22 kDa for CT-SGE (Figure 1B). In addition, we observed a ~ 10-kDa band in both, CT and FT, although the band showed stronger reactivity in FT-SGE (Figure 1C; Supplementary Figure S1). Mass spectrometry of the 10 kDa band revealed a total of 19 proteins. Specifically, eight proteins were unique to the CT-SGE, seven unique to the FT-SGE, and four were shared between both groups (Figure 1D). In the case of the ~25–22 kDa portion, a total of 30 proteins were identified, 11 proteins specific to CT-SGE, 13 in the FT-SGE and six were shared by both groups. The list of proteins can be found in Table 2.

**Figure 2:**
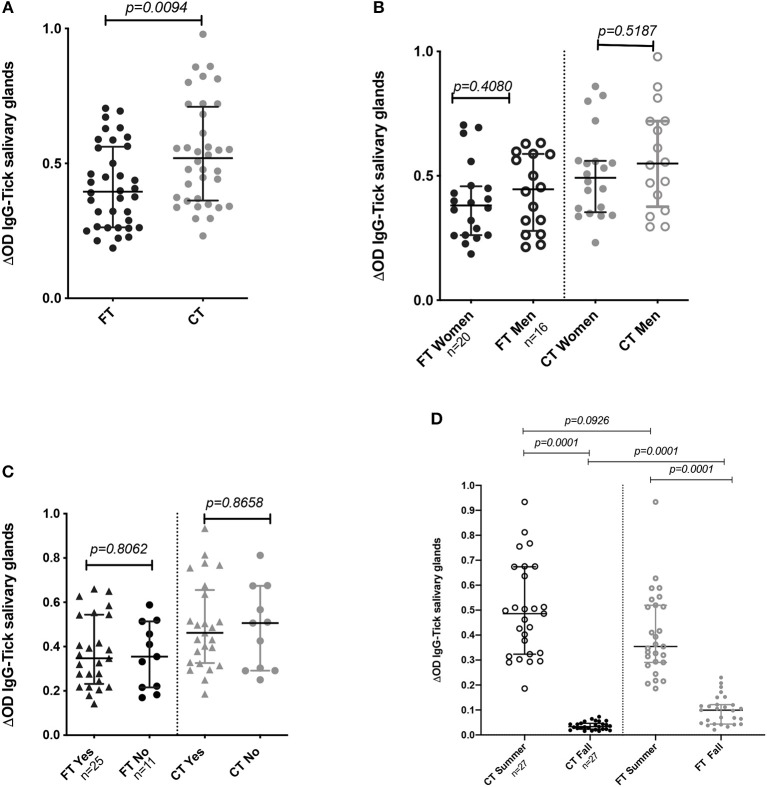
IgG Antibody levels in human samples tested by ELISA. **(A)** Total IgG antibody levels against FT-SGE and CT-SGE. **(B)** Comparison of anti SGE antibodies levels in women and men enrolled in summer 2018. **(C)** Comparison of IgG antibodies levels by use of repellent. **(D)** Comparison of antibody levels in Summer and Fall of 2018. Figure displaying the median with interquartile range.Significance evaluated by the Mann-Whitney test with a *p* < *0.05*.

**Table 2:** List of proteins found by mass spectrometry in the ~25–22 and 10 kDa gel area corresponding to the immunogenic bands in the immunoblot.

**Protein name**	**ID**	**MW**
**Only Field**		
**10 kDa Band**		
Putative m13 family peptidase^1^	A0A0C9RVU2	11,956
Putative ribosomal protein s18c	A0A0C9RZJ8	17798
Putative myosin regulatory light chain ef-hand protein	A0A0C9SCS4	19,957
**22–25 kDa Band**		
Putative lipocalin-5^1^	A0A0C9SE12	25,502
Putative polynucleotide kinase 3' phosphatase	A0A0C9RUF1	29,836
Putative endoplasmic reticulum glucose-regulated protein grp94/endoplasmin hsp90 family	A0A0C9SDH1	27,206
Serine protease inhibitor^2^	A0A0E9Y1R8	24,603
Putative cell cycle-associated protein	A0A0C9S1G1	22,560
40S ribosomal protein S3a	A0A0C9S283	30,205
Putative phosphoserine phosphatase	A0A0C9SBN8	26,146
**Only colony**		
**10 kDa Band**		
Putative vitellogenin	A0A0C9RSG8	15,853
Histone H4	A0A0C9QYX1	11,667
Putative calmodulin	A0A0C9QZX5	16,811
Putative lipocalin-3 1 lipocalin	A0A0C9SAU2	17,939
**22–25 kDa Band**		
Signal peptidase complex subunit 3	A0A0C9RR64	20,224
Spectrin alpha chain-like protein	B5M765	26,729
Putative chaperonin subunit	A0A0C9R1F3	23,782
Putative 26s protease regulatory subunit 4-like protein	A0A0C9SBM8	25,824
Putative secreted protein 94^1^	A0A0C9S5A9	23,779
**Shared**		
**10 kDa band**		
Ferritin	Q6WNX6	19,853
Putative 40s ribosomal protein s27a	A0A0C9SCH5	17,949
Putative a-macroglobulin receptor^2^	A0A0C9SC71	15,655
Alpha-2-macroglobulin^2^	B5M727	19,026
**22–25 kDa Band**		
Putative heme lipoprotein	A0A0C9RTH2	23,282
Putative vitellogenin-2	A0A0C9S1B0	20,974
Putative lipocal-1 14 lipocalin	A0A0C9SFF5	23,504
Actin	B5M764	21,136
Putative polyubiquitin	A0A0C9S1S7	25,821
Putative laminin g domain protein	A0A0C9S253	28,976

1 *Signal peptide*.

2 *Secreted protein*.

Since we used SGE and not saliva, we found both secreted and non-secreted proteins. Gene ontology revealed the function of 17 proteins, among those we could identify some with binding activity (*n* = 9), catalytic activity (*n* = 6), structural constituent of ribosome (*n* = 3), and lipid transporter activity (*n* = 1). From those proteins with an enzymatic activity we could identify hydrolases (*n* = 4), peptidases (*n* = 3), ferroxidase (*n* = 1), and kinases (*n* = 1). In addition, categorization by the biological process, we found proteins involved in cellular processes (*n* = 10), metabolic processes (*n* = 6), response to stimulus (*n* = 2) biological regulation (*n* = 2) and iron transport (*n* = 1). The only protein family found uniquely expressed as well as shared was the group of lipocalins, these proteins are found in the saliva of several arthropods and are abundant in tick saliva (48, 49). Our sequencing data revealed three lipocalins, one shared between tick groups and two individual lipocalins. BLAST analysis showed a 72% identity between a human lipocalin 2 protein (LCN2) (50) and the A0A0C9SE12 found in FT-SGE, while a 63% identity was found when comparing A0A0C9SAU2 from CT and only a 33% when comparing the shared A0A0C9SFF5. No other groups were found distributed among all categories analyzed (i.e., CT, FT, and shared.).

### Cytokine Gene Expression

**Figure 3:**
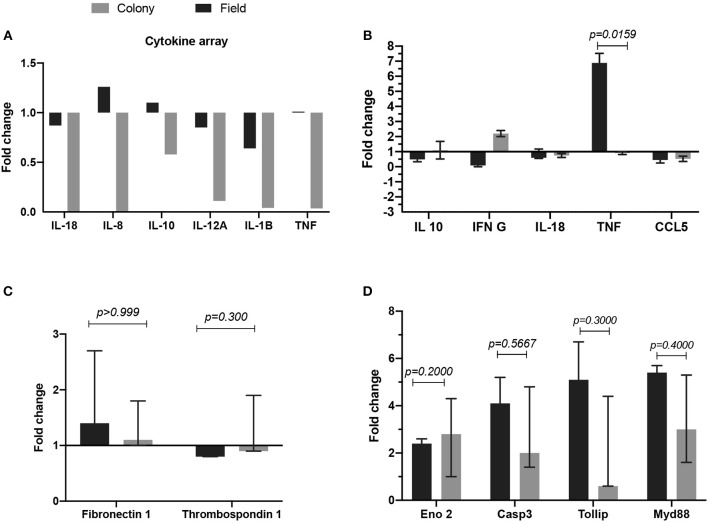
Comparison of fold change gene expression between cells treated with either FT-SGE or CT-SGE. **(A)** Cytokine array values in U937 cells. **(B)** qRT-PCR results U937 cells cytokine testing. **(C)** Fold change gene expression in endothelial cells, and **(D)** gene expression in neurons. Figure displaying the median with interquartile range. Significance was tested by the Mann-Whitney test with a *p* < *0.05*.

We tested the *in vitro* effect of CT-SGE and FT-SGE on human cells with the potential for interacting with the arthropod saliva during or after the blood feeding. Previous studies have shown the effect or arthropod saliva in the physiology of macrophages, endothelial cells and neurons (51). So, we explored the possibility that SGE from arthropods raised under different conditions may have a differential effect on host immunity. For this, we used a cytokine expression array and qRT-PCR to measure cytokine levels in the macrophage-like cell line U937 and exposed them to either CT-SGE or FT-SGE. Our results showed a slight upregulation (>1.0 fold) of IL-8, IL-10, and TNFα upon treatment with FT SGE (Figure 3A). Interestingly, the difference between SGE's was only significant in the TNFα levels (Mann-Whitney test, *p* = 0.0159) (Figure 3B). We did not find any significant differences in the expression of Fibronectin 1 and Thrombospondin 1 genes HUVEC cells (Figure 3C) or any of the genes tested in SH-SY5Y cells exposed to both groups of SGE, However, all these genes were upregulated in the SH-SY5Y upon incubation with the tick SGE (Figure 3D).

## Discussion

Arthropod saliva has recaptured attention in public health because of its involvement in the transmission of human pathogens. The use of antibodies against arthropod saliva as markers for bite exposure has been implemented to evaluate the risk of malaria and other mosquito-borne viruses with high reliability. In our study, we observed a significant reduction of anti-tick SGE antibodies from summer to fall suggesting a higher exposure to arthropod bites during the warmer months as observed previously. These results are in concordance to previous studies showing that antibodies against salivary proteins may be short-lived (52, 53). An unexpected finding was the higher antibody levels against the CT-SGE instead of the FT-SGE. Usually, colony arthropods are fed from the blood of one animal species for several generations. In this case, the colony ticks have been maintained in sheep for several years. Since several studies suggest that vertebrate host exert immunological pressure on salivary proteins, we speculate that the significantly higher concentration of salivary gland protein in the CT-SGE content may explain the observed results. Although the same concentration of SGE was used for all ELISAs, it is possible that the concentration of specific immunogenic proteins is higher in the CT-SGE than in the FT as revealed by our immunoblot. Our results suggest that the FT-SGE may have adapted to produce less concentration of highly immunogenic protein to induce fewer antibodies production and allow for longer feeding time. Importantly, immunogenic salivary proteins involved in blood feeding capable of inducing “good” antibody levels may be used as anti-arthropod vaccines (20, 22, 54).

A recent sialo-proteome study has reported up to 2,153 secreted proteins in *A. americanum* saliva and most of the proteins found in our study have been previously reported (20, 55). We found ten proteins only in FT-SGE, nine in CT-SGE only, and ten were shared between the two strains. Since we worked with SGE we found secreted and structural proteins. In the case of the FT SGE, three secreted proteins were uniquely found, a putative m13 family peptidase, a putative lipocalin-5 and a serine protease inhibitor. Another interesting protein found uniquely in the FT was a serine protease inhibitor (A0A0E9Y1R8). Among a wide range of functions, serine protease inhibitors are directly involved in the regulation of inflammation, blood clotting, wound healing, vasoconstriction. Also, several tick serine protease inhibitors are promising candidates for anti-tick vaccines (22, 56). In the case of the CT-SGE, we found an uncharacterized putative secreted protein 94 (A0A0C9S5A9) and a putative calmodulin (A0A0C9QZX5) among the proteins uniquely found in this tick group. Calmodulins are involved in calcium binding and previous studies describe a calmodulin involved in cellular signal transduction in *Haemaphysalis flava* (57).

Several proteins were found shared between the FT-SGE and the CT-SGE. Ferritin has previously found immunogenic in *A. americanum* salivary content (20). Ferritin has also been reported in salivary secretions from other ticks species where it plays important roles not only on iron metabolism and immunity but also as an anti-oxidant (58, 59). What may explain why it is important for both tick groups included in this study. Another important group of proteins found shared between tick groups were the α2-macroglobulin (α2M). These proteins are closely related to the C3, C4, and C5 components of the vertebrate complement system (60) and previous studies suggest a potential crosstalk of these molecules between vertebrates and invertebrates (61). The presence of α2Ms in the groups of shared proteins highlight the importance of immune defense during blood feedings since these groups of proteins are considered as early-acting innate immunity components, similar to opsonin (60). Also, a putative heme lipoprotein (A0A0C9RTH2) was found shared between CT and FT. A hem lipoprotein (HeLP) has been reported in the hemolymph of *Boophilus microplus*. This protein is able to bind up to 8 heme molecules and transports iron from the hemolymph to the tissues (62).

In this study, we explored for first the time the effect of tick saliva from different origin on neurons and endothelial cells *in vitro*. With this preliminary study, although no significant gene expression difference between strains was observed, our preliminary data suggest that both tick strains saliva could have an important impact in neuronal physiology, and further studies in this field are urgently needed. Although most of the cytokines measured were not significantly upregulated in concordance with previous studies suggesting an anti-inflammatory effect of tick saliva (63, 64), we observed a significantly higher expression of the pro-inflammatory cytokine TNFα (6-fold) in U937 cells when exposed to FT-SGE. Our hypothesis is that the presence of Lipocalin 5 in the FT-SGE may explain the discrepancies. In humans, a positive correlation of lipocalin-2 serum levels with serum TNFα levels was observed. There is a 72% identity between Lipocalin 2 and the putative Lipocalin 5 found that may explain why we observed higher TNF in macrophages exposed to this SGE. While the other two lipocalins, Lipocalin 3 found uniquely in SGE from CT and lipocalin 1 revealing only 63 and 33%, respectively. Lipocalins are abundant proteins in the saliva of both soft and hard ticks. Also, a lipocalin from *Ixodes ricinus* (LIR) was associated with modulation of inflammation (65). These lipocalins are associated regulating skin immune responses through scavenging bio-amines decreasing the sensation of pain during the blood-feeding process and have been associated with toxicosis, a toxic reaction against tick and mite bites (66). In addition, higher levels of TNFα, downregulate B cell responses and is associated with lower immunoglobulin production (67, 68) which might explain lower antibody levels after exposure to field tick saliva.

Increasing evidence suggesting a close interaction between the immune and the nervous system in control of pain sensation. During feeding, arthropods should modulate pain sensation to guarantee their repletion. MyD88 mediates the majority of the Toll-Like Receptors (TLR) signaling and modulating the production of inflammatory cytokines (69). TLRs members are also expressed in nervous cells including microglia and astrocytes which modulate pain and itch conditions. Our study showed more than 4-fold upregulation of these genes in cells treated with FT-SGE. Interestingly, Tollip was also found upregulated. Previous studies suggest that Tollip promotes neuronal apoptosis and inflammation (70). Its upregulation similar to the MyD88 may suggest a proinflammatory effect of SGE on SH-SY5Y. In addition, our results showed an upregulation in enolase 2 and caspase 3. Enolase 2 is induced upon inflammatory signaling and it associated with degradation of the extracellular matrix, and caspase 3 is associated with cell apoptosis (71), suggesting a proinflammatory response in the central nervous system. This is of importance since tick bites can also be associated with Tick paralysis, a non-infectious disease caused by specific protein in tick saliva that demonstrates the effect of salivary proteins on the vertebrate nervous system (72, 73). However, It is important to disclose that we used immature/undifferentiated SH-SY5Y and the effect of tick saliva on mature neurons may be different (74).

These preliminary findings could also suggest that different cells types are affected by tick saliva during bites and that these cells have a specific response, which requires more attention to better elucidate their role during inflammation and pathogenesis of neuronal tick-borne infectious diseases. Indeed, our results showing overall differences in the response against CT-SGE and FT-SGE could be due to the underlying genetic difference between tick strains. Previous studies suggest that genetic variation of *A. americanum* is highly affected by their abundance in the environment as well as their aggressiveness as ectoparasite feeding in multiple hosts and can be significant in ticks collected from different geographical locations (23, 75). They also suggest that the lack of specificity in host preference of *A. americanum* may be responsible for the significant genetic variation among tick populations (75, 76). Age-related differences in the tick salivary component may also explain the differences observed between the two tick groups included in this study (2, 58). Specifically, ticks from the colony have a specific age-matched by the time of molting. However, ticks collected in the field may have different age (in days) and represent a more heterogeneous mixture of individuals. Nonetheless, our results highlight the importance of conducting this type of studies with field collected ticks and not laboratory reared ticks as they more closely resemble the expected responses when in contact with a human host.

Although interesting, our study has several limitations. Working with SGE instead of pure saliva introduces more components that could cause an interaction during the measurement of antibody responses. We aim to deepen our findings using pure saliva extract from both, colony and field ticks. Also, the number of volunteers was limited and information about their travel history was not recorded, these factors may have an impact in the observed response arthropod saliva since previous or chronic exposure to salivary proteins may impact antibody profiles (77). We anticipate enrolling at least 200 participants in a larger study to confirm the results to the current study using whole tick saliva as the antigen instead of SGE. However, we consider that the results provided in this preliminary study may lead to important conclusions and future directions.

## Conclusion

This study shows that tick salivary gland protein content varies depending on the tick origin, indirectly showing that environmental conditions and probably host feeding preferences may have an impact on salivary gland content and immunogenicity. Also, our results suggest that salivary proteins from a tick can be used to measure the intensity of exposure to arthropod bites. However, not all salivary proteins are immunogenic, and this study reveals potential candidates to develop specific salivary biomarkers for *A. americanum* exposure.

## Data Availability

All datasets generated for this study in the sequencing data are included in the manuscript and the Supplementary Files. Other raw data will be available upon request sent to blondono@ksu.edu.

## Ethics Statement

The protocols followed in the study were reviewed and approved by IRB #1206 from Kansas State University. Blood samples were only collected from individuals agree to participate in the study and that had signed an informed consent. Dried blood spots in filter paper Whatman 903 were obtained from 36 healthy donors in the summer (May–June), and only 27 of those could be followed in the fall (September–November). Blood samples were obtained from adult volunteers residing in Manhattan, Kansas. At enrollment, tested individuals were provided with a questionnaire for an information about age, gender, use of repellant and outdoor activities.

## Author Contributions

LM-R: study design, sample collection, sample processing, analysis, writing, and reviewing. LM-C: study design, human cells *in vitro* testing, analysis, writing, and reviewing. BB: study design, sample collection, sample processing, analysis, writing, and reviewing. SM: writing and reviewing. LZ: analysis, writing, and reviewing. BL-R: study design, sample collection, analysis, writing, and reviewing.

### Conflict of Interest Statement

The authors declare that the research was conducted in the absence of any commercial or financial relationships that could be construed as a potential conflict of interest.

## Supplementary Material

Supplementary Table S1 Primer sequence of genes associated with injury/damage and immunity used in this study.

Supplementary Figure S1 Technical replicates of immunoblot using human samples from healthy volunteers against FT and CT.

## References

[1] Brites-Neto J Duarte KM Martins TF. Tick-borne infections in human and animal population worldwide. Vet World. (2015) 8:301–15. 10.14202/vetworld.2015.301-315 27047089 PMC4774835

[2] Simo L Kazimirova M Richardson J Bonnet SI. The essential role of tick salivary glands and saliva in tick feeding and pathogen transmission. Front Cell Infect Microbiol. (2017) 7:281. 10.3389/fcimb.2017.00281 28690983 PMC5479950

[3] Nuttall PA. Wonders of tick saliva. Ticks Tick Borne Dis. (2019) 10:470–81. 10.1016/j.ttbdis.2018.11.005 30459085

[4] Titus RG Bishop JV Mejia JS. The immunomodulatory factors of arthropod saliva and the potential for these factors to serve as vaccine targets to prevent pathogen transmission. Parasite Immunol. (2006) 28:131–41. 10.1111/j.1365-3024.2006.00807.x 16542315

[5] Kotal J Langhansova H Lieskovska J Andersen JF Francischetti IM Chavakis T. Modulation of host immunity by tick saliva. J Proteomics. (2015) 128:58–68. 10.1016/j.jprot.2015.07.005 26189360 PMC4619117

[6] Perner J Kropackova S Kopacek P Ribeiro JMC. Sialome diversity of ticks revealed by RNAseq of single tick salivary glands. PLoS Negl Trop Dis. (2018) 12:e0006410. 10.1371/journal.pntd.0006410 29652888 PMC5919021

[7] Kazimirova M Thangamani S Bartikova P Hermance M Holikova V Stibraniova I. Tick-borne viruses and biological processes at the tick-host-virus interface. Front Cell Infect Microbiol. (2017) 7:339. 10.3389/fcimb.2017.00339 28798904 PMC5526847

[8] Hermance ME Thangamani S. Tick saliva enhances powassan virus transmission to the host, influencing its dissemination and the course of disease. J Virol. (2015) 89:7852–60. 10.1128/JVI.01056-15 25995246 PMC4505606

[9] Nuttall PA Tick saliva and its role in pathogen transmission. Wien Klin Wochenschr. (2019) 10:1–12. 10.1007/s00508-019-1500-y PMC10118219 31062185

[10] Schuijt TJ Hovius JW van Burgel ND Ramamoorthi N Fikrig E van Dam AP. The tick salivary protein Salp15 inhibits the killing of serum-sensitive Borrelia burgdorferi sensu lato isolates. Infect Immun. (2008) 76:2888–94. 10.1128/IAI.00232-08 18426890 PMC2446733

[11] Andrade BB Teixeira CR Barral A Barral-Netto M. Haematophagous arthropod saliva and host defense system: a tale of tear and blood. An Acad Bras Cienc. (2005) 77:665–93. 10.1590/S0001-37652005000400008 16341443

[12] Scholl DC Embers ME Caskey JR Kaushal D Mather TN Buck WR. Immunomodulatory effects of tick saliva on dermal cells exposed to Borrelia burgdorferi, the agent of Lyme disease. Parasit Vectors. (2016) 9:394. 10.1186/s13071-016-1638-7 27391120 PMC4938952

[13] Wikel S. Ticks and tick-borne pathogens at the cutaneous interface: host defenses, tick countermeasures, a suitable environment for pathogen establishment. Front Microbiol. (2013) 4:337. 10.3389/fmicb.2013.00337 24312085 PMC3833115

[14] Londono-Renteria B Cardenas JC Giovanni JE Cardenas L Villamizar P Rolon J. *Aedes aegypti* anti-salivary gland antibody concentration and dengue virus exposure history in healthy individuals living in an endemic area in Colombia. Biomedica. (2015) 35:572–81. 10.7705/biomedica.v35i4.2530 26844447

[15] Londono-Renteria B Patel JC Vaughn M Funkhauser S Ponnusamy L Grippin C. Long-lasting permethrin-impregnated clothing protects against mosquito bites in outdoor workers. Am J Trop Med Hyg. (2015) 93:869–74. 10.4269/ajtmh.15-0130 26195460 PMC4596613

[16] Lane RS Moss RB Hsu YP Wei T Mesirow ML Kuo MM. Anti-arthropod saliva antibodies among residents of a community at high risk for Lyme disease in California. Am J Trop Med Hyg. (1999) 61:850–9. 10.4269/ajtmh.1999.61.850 10586924

[17] Huang YS Higgs S Vanlandingham DL. Arbovirus-mosquito vector-host interactions and the impact on transmission and disease pathogenesis of arboviruses. Front Microbiol. (2019) 10:22. 10.3389/fmicb.2019.00022 30728812 PMC6351451

[18] Nuttall PA Labuda M. Tick-host interactions: saliva-activated transmission. Parasitology. (2004) 129 (Suppl. 1):S177–89. 10.1017/S0031182004005633 15938511

[19] Ferreira BR Szabo MJ Cavassani KA Bechara GH Silva JS Antigens from Rhipicephalus sanguineus ticks elicit potent cell-mediated immune responses in resistant but not in susceptible animals. Vet Parasitol. (2003) 115:35–48. 10.1016/S0304-4017(03)00190-0 12860066

[20] Radulovic ZM Kim TK Porter LM Sze SH Lewis L Mulenga A. A 24–48 h fed Amblyomma americanum tick saliva immuno-proteome. BMC Genomics. (2014) 15:518. 10.1186/1471-2164-15-518 24962723 PMC4099483

[21] Ribeiro JM. How ticks make a living. Parasitol Today. (1995) 11:91–3. 10.1016/0169-4758(95)80162-6 15275359

[22] Sprong H Trentelman J Seemann I Grubhoffer L Rego RO Hajdusek O. ANTIDotE: anti-tick vaccines to prevent tick-borne diseases in Europe. Parasit Vectors. (2014) 7:77. 10.1186/1756-3305-7-77 24559082 PMC3933510

[23] Childs JE Paddock CD. The ascendancy of Amblyomma americanum as a vector of pathogens affecting humans in the United States. Annu Rev Entomol. (2003) 48:307–37. 10.1146/annurev.ento.48.091801.112728 12414740

[24] Barrett AW Noden BH Gruntmeir JM Holland T Mitcham JR Martin JE. County scale distribution of *Amblyomma americanum* (Ixodida: Ixodidae) in Oklahoma: addressing local deficits in tick maps based on passive reporting. J Med Entomol. (2015) 52:269–73. 10.1093/jme/tju026 26336311

[25] Raghavan RK Peterson AT Cobos ME Ganta R Foley D. Current and future distribution of the Lone Star Tick, *Amblyomma americanum* (L.) (Acari: Ixodidae) in North America. PLoS ONE. (2019) 14:e0209082. 10.1371/journal.pone.0209082 30601855 PMC6314611

[26] Mani RJ Metcalf JA Clinkenbeard KD. *Amblyomma americanum* as a bridging vector for human infection with *Francisella tularensis*. PLoS ONE. (2015) 10:e0130513. 10.1371/journal.pone.0130513 26121137 PMC4486451

[27] Crispell G Commins SP Archer-Hartman SA Choudhary S Dharmarajan G Azadi P. Discovery of alpha-gal-containing antigens in north american tick species believed to induce red meat allergy. Front Immunol. (2019) 10:1056. 10.3389/fimmu.2019.01056 31156631 PMC6533943

[28] Monzon JD Atkinson EG Henn BM Benach JL. Population and evolutionary genomics of *Amblyomma americanum*, an expanding arthropod disease vector. Genome Biol Evol. (2016) 8:1351–60. 10.1093/gbe/evw080 27190204 PMC4898797

[29] Centers for Disease Control and Prevention. Tickborne diseases of the United States. (2019). Available online at: https://www.cdc.gov/ticks/tickbornediseases/tickID.html

[30] Sonenshine DE Nicholson WL. Ticks (Ixodida). In: Mullen GR Durden LA, editors. Medical and Veterinary Entomology. Statesboro, GA: Academic Press (2002). p. 517–58.

[31] Londono-Renteria BL Eisele TP Keating J James MA Wesson DM. Antibody response against *Anopheles albimanus* (Diptera: Culicidae) salivary protein as a measure of mosquito bite exposure in Haiti. J Med Entomol. (2010) 47:1156–63. 10.1603/ME09240 21175067

[32] Londono-Renteria B Drame PM Weitzel T Rosas R Gripping C Cardenas JC. *An. gambiae* gSG6-P1 evaluation as a proxy for human-vector contact in the Americas: a pilot study. Parasit Vectors. (2015) 8:533. 10.1186/s13071-015-1160-3 26464073 PMC4605097

[33] Conway MJ Londono-Renteria B Troupin A Watson AM Klimstra WB Fikrig E. *Aedes aegypti* D7 saliva protein inhibits dengue virus infection. PLoS Negl Trop Dis. (2016) 10:e0004941. 10.1371/journal.pntd.0004941 27632170 PMC5025043

[34] Vogt MB Lahon A Arya RP Kneubehl AR Spencer Clinton JL Paust S. Mosquito saliva alone has profound effects on the human immune system. PLoS Negl Trop Dis. (2018) 12:e0006439. 10.1371/journal.pntd.0006439 29771921 PMC5957326

[35] T.Nascimento GD Vieira PS Cogo SC Dias-Netipanyj MF Franca Junior N Camara DAD Antitumoral effects of *Amblyomma sculptum* Berlese saliva in neuroblastoma cell lines involve cytoskeletal deconstruction and cell cycle arrest. Rev Bras Parasitol Vet. (2019) 28:126–33. 10.1590/s1984-296120180098 30785557

[36] Shipley MM Mangold CA Kuny CV Szpara ML. Differentiated human SH-SY5Y cells provide a reductionist model of herpes simplex virus 1 neurotropism. J Virol. (2017) 91:e00958-17. 10.1128/JVI.00958-17 28956768 PMC5686721

[37] Gokyu M Kobayashi H Nanbara H Sudo T Ikeda Y Suda T. Thrombospondin-1 production is enhanced by Porphyromonas gingivalis lipopolysaccharide in THP-1 cells. PLoS ONE. (2014) 9:e115107. 10.1371/journal.pone.0115107 25501558 PMC4264871

[38] Tabata S Ikeda R Yamamoto M Shimaoka S Mukaida N Takeda Y. Thymidine phosphorylase activates NFkappaB and stimulates the expression of angiogenic and metastatic factors in human cancer cells. Oncotarget. (2014) 5:10473–85. 10.18632/oncotarget.2242 25350954 PMC4279387

[39] Pimentel-Nunes P Goncalves N Boal-Carvalho I Afonso L Lopes P Roncon-Albuquerque R Jr. Decreased Toll-interacting protein and peroxisome proliferator-activated receptor gamma are associated with increased expression of Toll-like receptors in colon carcinogenesis. J Clin Pathol. (2012) 65:302–8. 10.1136/jclinpath-2011-200567 22228906

[40] Gu H Jiao Y Yu X Li X Wang W Ding L. Resveratrol inhibits the IL-1beta-induced expression of MMP-13 and IL-6 in human articular chondrocytes via TLR4/MyD88-dependent and -independent signaling cascades. Int J Mol Med. (2017) 39:734–40. 10.3892/ijmm.2017.2885 28204817

[41] Du M Wang X Tan X Li X Huang D Huang K. Nkx2-5 is expressed in atherosclerotic plaques and attenuates development of atherosclerosis in apolipoprotein E-deficient mice. J Am Heart Assoc. (2016) 5:e004440. 10.1161/JAHA.116.004440 27993833 PMC5210424

[42] Li Y Chen R Bowden M Mo F Lin YY Gleave M. Establishment of a neuroendocrine prostate cancer model driven by the RNA splicing factor SRRM4. Oncotarget. (2017) 8:66878–88. 10.18632/oncotarget.19916 28978002 PMC5620142

[43] Feng M Kang M He F Xiao Z Liu Z Yao H. Plasma interleukin-37 is increased and inhibits the production of inflammatory cytokines in peripheral blood mononuclear cells in systemic juvenile idiopathic arthritis patients. J Transl Med. (2018) 16:277. 10.1186/s12967-018-1655-8 30305171 PMC6180625

[44] Wang B Wei G Liu B Zhou X Xiao H Dong N. The role of high mobility group box 1 protein in interleukin-18-induced myofibroblastic transition of valvular interstitial cells. Cardiology. (2016) 135:168–78. 10.1159/000447483 27395056

[45] Kawka E Witowski J Fouqet N Tayama H Bender TO Catar R. Regulation of chemokine CCL5 synthesis in human peritoneal fibroblasts: a key role of IFN-gamma. Mediators Inflamm. (2014) 2014:590654. 10.1155/2014/590654 24523572 PMC3913084

[46] Plotnikova MA Klotchenko SA Vasin AV. Development of a multiplex quantitative PCR assay for the analysis of human cytokine gene expression in influenza A virus-infected cells. J Immunol Methods. (2016) 430:51–5. 10.1016/j.jim.2016.01.005 26772136

[47] Kim S Kim YK Lee H Cho JE Kim HY Uh Y. Interferon gamma mRNA quantitative real-time polymerase chain reaction for the diagnosis of latent tuberculosis: a novel interferon gamma release assay. Diagn Microbiol Infect Dis. (2013) 75:68–72. 10.1016/j.diagmicrobio.2012.09.015 23102550

[48] Sanchez D Ganfornina MD Gutierrez G Marin A. Exon-intron structure and evolution of the Lipocalin gene family. Mol Biol Evol. (2003) 20:775–83. 10.1093/molbev/msg079 12679526

[49] Paesen GC Adams PL Nuttall PA Stuart DL. Tick histamine-binding proteins: lipocalins with a second binding cavity. Biochim Biophys Acta. (2000) 1482:92–101. 10.1016/S0167-4838(00)00168-0 11058751

[50] Wolk K Wenzel J Tsaousi A Witte-Handel E Babel N Zelenak C. Lipocalin-2 is expressed by activated granulocytes and keratinocytes in affected skin and reflects disease activity in acne inversa/hidradenitis suppurativa. Br J Dermatol. (2017) 177:1385–93. 10.1111/bjd.15424 28256718

[51] Fontaine A Diouf I Bakkali N Misse D Pages F Fusai T. Implication of haematophagous arthropod salivary proteins in host-vector interactions. Parasit Vectors. (2011) 4:187. 10.1186/1756-3305-4-187 21951834 PMC3197560

[52] Londono-Renteria B Cardenas JC Cardenas LD Christofferson RC Chisenhall DM Wesson DM. Use of anti-Aedes aegypti salivary extract antibody concentration to correlate risk of vector exposure and dengue transmission risk in Colombia. PLoS ONE. (2013) 8:e81211. 10.1371/journal.pone.0081211 24312537 PMC3846924

[53] Fontaine A Pascual A Orlandi-Pradines E Diouf I Remoue F Pages F. Relationship between exposure to vector bites and antibody responses to mosquito salivary gland extracts. PLoS ONE. (2011) 6:e29107. 10.1371/journal.pone.0029107 22195000 PMC3237593

[54] Rego ROM Trentelman JJA Anguita J Nijhof AM Sprong H Klempa B. Counterattacking the tick bite: towards a rational design of anti-tick vaccines targeting pathogen transmission. Parasit Vectors. (2019) 12:229. 10.1186/s13071-019-3468-x 31088506 PMC6518728

[55] Karim S Ribeiro JM. An insight into the sialome of the lone star tick, *Amblyomma americanum*, with a glimpse on its time dependent gene expression. PLoS ONE. (2015) 10:e0131292. 10.1371/journal.pone.0131292 26131772 PMC4489193

[56] Sugino M Imamura S Mulenga A Nakajima M Tsuda A Ohashi K. A serine proteinase inhibitor (serpin) from ixodid tick Haemaphysalis longicornis; cloning and preliminary assessment of its suitability as a candidate for a tick vaccine. Vaccine. (2003) 21:2844–51. 10.1016/S0264-410X(03)00167-1 12798626

[57] Liu L Cheng TY He XM. Proteomic profiling of the midgut contents of *Haemaphysalis flava*. Ticks Tick Borne Dis. (2018) 9:490–5. 10.1016/j.ttbdis.2018.01.008 29371124

[58] Tirloni L Reck J Terra RM Martins JR Mulenga A Sherman NE. Proteomic analysis of cattle tick Rhipicephalus (Boophilus) microplus saliva: a comparison between partially and fully engorged females. PLoS ONE. (2014) 9:e94831. 10.1371/journal.pone.0094831 24762651 PMC3998978

[59] Lewis LA Radulovic ZM Kim TK Porter LM Mulenga A. Identification of 24h ixodes scapularis immunogenic tick saliva proteins. Ticks Tick Borne Dis. (2015) 6:424–34. 10.1016/j.ttbdis.2015.03.012 25825233 PMC4415496

[60] Sottrup-Jensen L Stepanik TM Kristensen T Lonblad PB Jones CM Wierzbicki DM. Common evolutionary origin of alpha 2-macroglobulin and complement components C3 and C4. Proc Natl Acad Sci USA. (1985) 82:9–13. 10.1073/pnas.82.1.9 2578664 PMC396960

[61] Londono-Renteria B Grippin C Cardenas JC Troupin A Colpitts TM. Human C5a protein participates in the mosquito immune response against dengue virus. J Med Entomol. (2016) 53:505–12. 10.1093/jme/tjw003 26843451 PMC4892811

[62] Maya-Monteiro CM Daffre S Logullo C Lara FA Alves EW Capurro ML. HeLp, a heme lipoprotein from the hemolymph of the cattle tick, *Boophilus microplus*. J Biol Chem. (2000) 275:36584–9. 10.1074/jbc.M007344200 10964932

[63] Tian Y Chen W Mo G Chen R Fang M Yedid G. An immunosuppressant peptide from the hard tick *Amblyomma variegatum*. Toxins. (2016) 8:E133. 10.3390/toxins8050133 27153086 PMC4885048

[64] Ramachandra RN Wikel SK. Modulation of host-immune responses by ticks (Acari: Ixodidae): effect of salivary gland extracts on host macrophages and lymphocyte cytokine production. J Med Entomol. (1992) 29:818–26. 10.1093/jmedent/29.5.818 1404261

[65] Beaufays J Adam B Decrem Y Prevot PP Santini S Brasseur R. Ixodes ricinus tick lipocalins: identification, cloning, phylogenetic analysis and biochemical characterization. PLoS ONE. (2008) 3:e3941. 10.1371/journal.pone.0003941 19096708 PMC2601031

[66] Mans BJ Louw AI Neitz AW. The major tick salivary gland proteins and toxins from the soft tick, *Ornithodoros savignyi*, are part of the tick Lipocalin family: implications for the origins of tick toxicoses. Mol Biol Evol. (2003) 20:1158–67. 10.1093/molbev/msg126 12777525

[67] Frasca D Romero M Diaz A Alter-Wolf S Ratliff M Landin AM. A molecular mechanism for TNF-alpha-mediated downregulation of B cell responses. J Immunol. (2012) 188:279–86. 10.4049/jimmunol.1003964 22116831 PMC3700394

[68] Rieckmann P Tuscano JM Kehrl JH. Tumor necrosis factor-alpha (TNF-alpha) and interleukin-6 (IL-6) in B-lymphocyte function. Methods. (1997) 11:128–32. 10.1006/meth.1996.0396 8990098

[69] Liu XJ Liu T Chen G Wang B Yu XL Yin C. TLR signaling adaptor protein MyD88 in primary sensory neurons contributes to persistent inflammatory and neuropathic pain and neuroinflammation. Sci Rep. (2016) 6:28188. 10.1038/srep28188 27312666 PMC4911580

[70] Li M Feng B Wang L Guo S Zhang P Gong J. Tollip is a critical mediator of cerebral ischaemia-reperfusion injury. J Pathol. (2015) 237:249–62. 10.1002/path.4565 26011492

[71] Haque A Ray SK Cox A Banik NL. Neuron specific enolase: a promising therapeutic target in acute spinal cord injury. Metab Brain Dis. (2016) 31:487–95. 10.1007/s11011-016-9801-6 26847611 PMC4864119

[72] Borawski K Pancewicz S Czupryna P Zajkowska J Moniuszko-Malinowska A. Tick paralysis. Przegl Epidemiol. (2018) 72:17–24. 29667376

[73] Pienaar R Neitz AWH Mans BJ. Tick paralysis: solving an enigma. Vet Sci. (2018) 5:E53. 10.3390/vetsci5020053 29757990 PMC6024606

[74] Shipley MM Mangold CA Szpara ML. Differentiation of the SH-SY5Y human neuroblastoma cell line. J Vis Exp. (2016) 53193. 10.3791/53193 PMC4828168 26967710

[75] Trout RT Steelman CD Szalanski AL. Population genetics of *Amblyomma americanum* (Acari: Ixodidae) collected from Arkansas. J Med Entomol. (2010) 47:152–61. 10.1093/jmedent/47.2.152 20380295

[76] Barrett LG Thrall PH Burdon JJ Linde CC. Life history determines genetic structure and evolutionary potential of host-parasite interactions. Trends Ecol Evol. (2008) 23:678–85. 10.1016/j.tree.2008.06.017 18947899 PMC2653456

[77] Cardenas JC Drame PM Luque-Burgos KA Berrio JD Entrena-Mutis E Gonzalez MU. IgG1 and IgG4 antibodies against *Aedes aegypti* salivary proteins and risk for dengue infections. PLoS ONE. (2019) 14:e0208455. 10.1371/journal.pone.0208455 30601814 PMC6314615

