# Supplementary material for: Differential Tick Salivary Protein Profiles and Human Immune Responses to Lone Star Ticks (Amblyomma americanum) From the Wild vs. a Laboratory Colony
Source: Front Immunol. 2019 Aug 28;10:1996. doi: 10.3389/fimmu.2019.01996 (PMC6724717; doi:10.3389/fimmu.2019.01996)
Supplement: Supplementary Table S1 — Primer sequence of genes associated with injury/damage and immunity used in this study. [file Table_1.docx]

Supplementary Table 1: Primer sequence of genes associated with injury/damage and immunity used in this study.

| **Gene** | **Primer sense** | **Primer antisense** | **Reference** |
| --- | --- | --- | --- |
| CXCR4 | 5-AGG TGC TGA AAT CAA CCC AC-3 | 5-CGT GGAACGTTTTTCCTG TT-3 | Azariadis 2017 |
| TOLLIP | 5-TCCCCGCTGGAATAAGGT-3 | 5-CGTCCATGGAGAAGGCTCT | Pimentel-Nunes, 2012 |
| MYD88 | 5-CACTCAGCCTCTCTCCAGGT-3 | 5-AGTCTTCAGGGCAGGGACA-3 | Gu, 2017 |
| CCL2 | 5-CTTCTGTGCCTGCTGCTCAT-3 | 5-CG GAGTTTGGGTTTGCTTGTC-3 | Zhang, 2015 |
| IL-22 | 5-AGGCTCAGCAACAGGCTAAG-3 | 5-TTTGCTCTGGTCAAATGCAG-3 | Muls 2017 |
| CASPASE3 | 5-CATGGAAGCGAATCAATGGACT-3 | 5-CTGTACCAGACCGAGATGTCA-3 | Du, 2016 |
| ENOLASE2 | 5-CCGGGAACTCAGACCTCATC-3 | 5-CTCTGCACCTAGTCGCATGG-3 | Li, 2017 |
| Fibronectin 1 | 5-GAATAAGCTGTACCATCGCA-3 | 5-GGTGTCACCAATCTTGTAGG-3 | Tabata, 2014 |
| Thrombospondin 1 | 5-ATGGAATTGGTGATGCCTGTG-3 | 5-ACTGAGCTGGGTTGTAATGGAATG-3 | Gokyu, 2014 |
