# Supplementary figures and images for: Differential Tick Salivary Protein Profiles and Human Immune Responses to Lone Star Ticks (Amblyomma americanum) From the Wild vs. a Laboratory Colony
Source: Front Immunol. 2019 Aug 28;10:1996. doi: 10.3389/fimmu.2019.01996 (PMC6724717; doi:10.3389/fimmu.2019.01996)

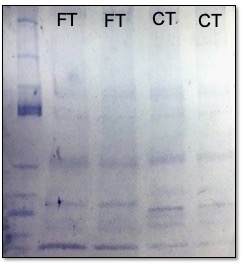

Supplement: Supplementary Figure S1 — Technical replicates of immunoblot using human samples from healthy volunteers against FT and CT. [file Image_1.JPEG]
